# Regional variations in the growth of Saudi children and adolescents

**DOI:** 10.4103/0256-4947.55163

**Published:** 2009

**Authors:** Mohammad El Mouzan, Peter Foster, Abdullah Al Herbish, Abdullaha Al Salloum, Ahmad Al Omer, Mansour Alqurashi, Tatjana Kecojevic

**Affiliations:** a From the Department of Pediatrics, King Saud University, Riyadh, Saudi Arabia; b From the Manchester University, Manchester, United Kingdom; c From the Riyadh Medical Complex, Riyadh, Saudi Arabia; d From the Department of Pediatrics, Al Yamamah Hospital, Riyadh, Saudi Arabia

## Abstract

**BACKGROUND AND OBJECTIVES:**

No previous study has provided a detailed description of regional variations of growth within the various regions of Saudi Arabia. Thus, we sought to demonstrate differences in growth of children and adolescents in different regions.

**SUBJECTS AND METHODS:**

The 2005 Saudi reference was based on a cross-sectional representative sample of the Saudi population of healthy children and adolescents from birth to 18 years of age. Body measurements of the length, stature, weight, head circumference and calculation of the BMI were performed according to standard recommendations. Percentile construction and smoothing were performed using the LMS (lambda, mu and sigma) methodology, followed by transformation of all individual measurements into standard deviation scores. Factors such as weight for age, height for age, weight for height, and head circumference for children from birth to 3 years, stature for age, head circumference and body mass index for children between 2-18 years of age were assessed. Subsequently, variations in growth between the three main regions in the north, southwest, and center of Saudi Arabia were calculated, with the Bonferroni: method used to assess the significance of differences between regions.

**RESULTS:**

There were significant differences in growth between regions that varied according to age, gender, growth parameter and region. The highest variation was found between children and adolescents of the southwestern region and those of the other two regions The regression lines for all growth parameters in children <3 years of age were significantly different from one region to another reaching – 0.65 standard deviation scores for the southwestern regions (*P*=.001). However, the difference between the northern and central regions were not significant for the head circumference and for weight for length. For older children and adolescents a significant difference was found in all parameters except between the northern and central regions in BMI in girls and head circumference in boys. Finally, the difference in head circumference of girls between southwestern and northern regions was not significant. Such variation affected all growth parameters for both boys and girls.

**CONCLUSION:**

Regional variations in growth need to be taken into consideration when assessing the growth of Saudi children and adolescents.

Growth indicators in the form of weight, length, stature, head circumference, weight for length and body mass index (BMI) for age are important tools for the assessment of growth of children and adolescents. Variations in growth are well known between countries as well as within the same country.^1^,^2^ In Saudi Arabia, previous studies based on the growth of children and adolescents have been conducted and comparisons with other countries have been made.^3^–^7^ However, to our knowledge, none of these studies provided a detailed description of regional variations of growth within Saudi Arabia. In this report, we analyze regional variations in the most recent and comprehensive growth study in Saudi children and adolescents.^8^

## SUBJECTS AND METHODS

The design and methodology of the Saudi reference, which is the basis of this analysis of regional variations, have been reported in detail elsewhere.^8^ Briefly, standard guidelines and criteria were followed in the determination of the sample size.^9^ The study sample was selected by a multistage probability sampling procedure from a stratified listing based on the population census available at the time of the study. Accordingly, the sample was a representation of all the socioeconomic strata. The majority of young children were on both breast milk and formula feedings. A pilot study was designed to test all components of the project before data collection. A training workshop was conducted for the members of the field teams in each of the 13 regions of Saudi Arabia. Data collection was made by house-to-house visits where a survey questionnaire, clinical examination and body measurements were completed by primary care physicians and nurses.

The lambda, mu and sigma (LMS), statistical methodology was used to construct and smooth the growth charts.^10^–^12^ Growth data collected from three regions from the north (Hail Jouf, Northern Borders) were compared with data collected from two regions from the southwest (Gizan and Aseer) and with two regions in the middle of the country (Riyadh and Qassim). The final LMS model was used to transform all individual measurements into standard deviation scores (SDS). Three steps were performed: In the first step, a separate cubic regression curve, where the response (“*y*-variable”) is the SDS and the covariate (“*x*-variable”) is age, was fitted to the data in each of the three categories of regions. These regression lines describe how the mean SDS of a given measurement changes with age in each region. The fit of the three cubic regression curves was then compared with the fit of three quadratic regression curves. If the difference in fits was not statistically significant, then the quadratic models were accepted and they were then compared with three linear regression curves and so on until the simplest model that might be fitted was three different horizontal lines. The three final regression lines were then plotted to provide a graphical description of the differences between regions. The second step was to statistically test the fit of the model involving three separate regression lines with the fit of the model based on a single common regression line. The standard F test was used to determine the statistical significance of the fit of the above-mentioned models and if the *P* value was small (less than .05, or less than .01 if the sample size was large); there were significant differences in how the mean SDS of a given measurement of a given age group and a given sex change with age. The third step, after a finding a significant difference, the same methodology was used to compare the difference between pairs of regions in turn to see whether they are significantly different from each other. To account for carrying out multiple comparisons between pairs of regions for a particular measure, the Bonferroni method which divides the total significance level into three (i.e., .01/3), was used. Accordingly, the difference between pairs of regions would be significant if the *P* value was less than .003.

## RESULTS

### Number of children and adolescents

The number of children and adolescents used in the analysis was 7723 (3912 boys and 3811 girls), 5072 (2590 boys and 2482 girls) and 6336 (3209 boys and 3127 girls) from the central, southwestern and northern regions, respectively. The number of children from 0 to 3 years were 4331, 1653, 2099 and those between 2 to 18 years were 3525, 3413, 4174 in the central, southwestern and northern regions, respectively. The difference between the total number of children and the sum of the two age groups is caused by the overlap 2-3-year age group.

### General interpretation

**Figure 1:**
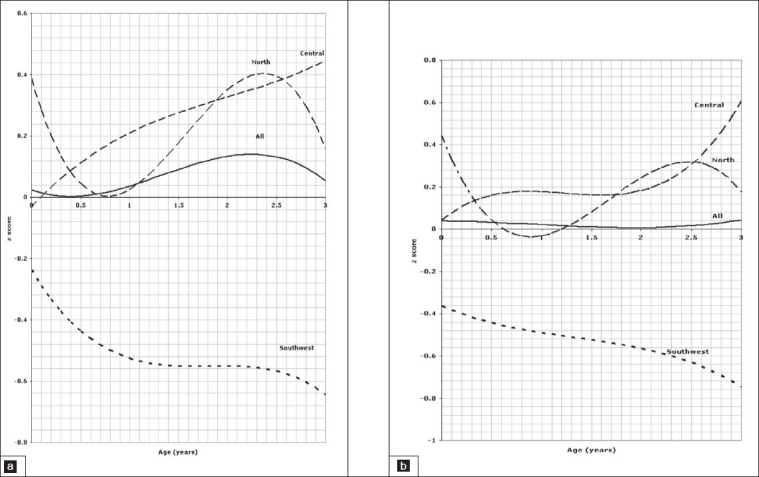
Weight of children aged 0 to 3 years, 1a for boys, 1b for girls.

The reference line for the average of the three regions is indicated on the figures as “all”. The units are standard deviation scores based on all of the data used to fit the LMS model for a given measurement in order to examine the nature of the differences of that measurement over the age range. The figures clearly highlight the differences between the regions over the age range. Where there is no significant difference between pairs of regions (for example north-central), the curves are fairly close, but for pairs of regions that are significantly different they are well separated. The *P* values in this analysis indicate whether or not there are overall significant differences of a given parameter between regions. For example, in Figure 1a, it is clear that the regression line of the *z* score of weight for boys aged birth to 3 years in the southwest was negative relative not only to the curves of the central and north, but also to the curve of “all” regions which is the reference line, indicating a lower weight than any other region, reaching a z score of – .65 below the regression line. On the other hand, the regression line for weight of children in the north and central regions are all positive relative to the reference line reflecting better weight of children living in these regions. The same method of interpretation applies to all figures.

### Birth to 3-years old

**Figure 2:**
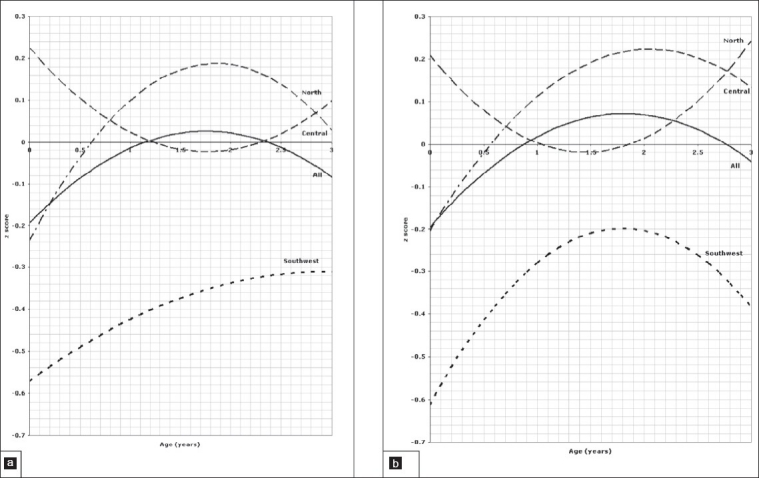
Length of children aged 0 to 3 years, 2a for boys, 2b for girls.

**Figure 3:**
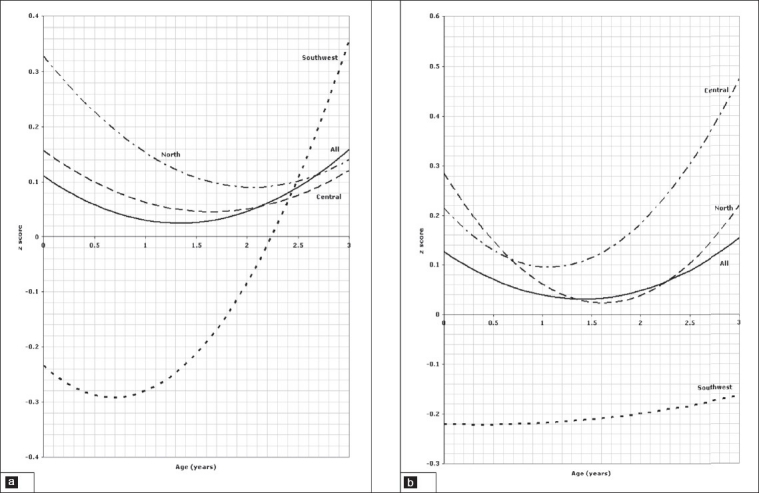
Head circumference of children aged 0 to 3 years, 3a for boys, 3b for girls.

**Figure 4:**
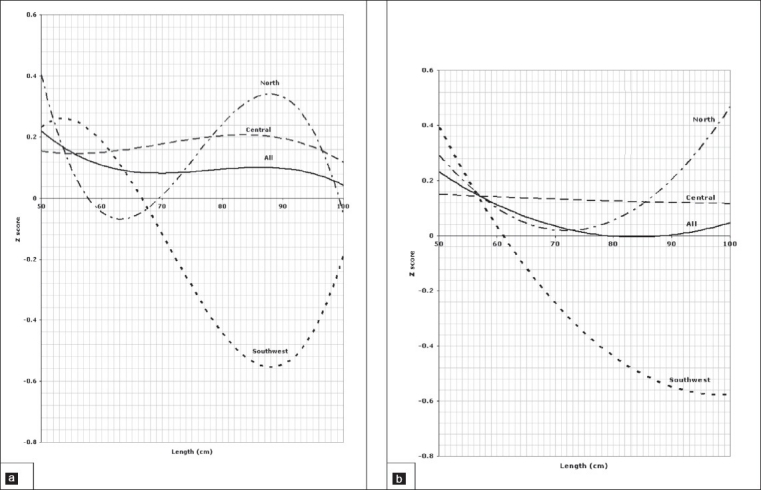
Weight for length of children aged 0 to 3 years, 4a for boys, 4b for girls.

Comparison of the weight for age *z* scores between regions in the age group between birth and 3 years is depicted in Figure 1a for boys and Figure 1b for girls indicating a significant difference between all three regions (*P*=.001). The pattern of regional variation in the *l*ength for age z scores in the age group between 0 and 3 years is presented in Figure 2a for boys and Figure 2b for girls which show again a significant difference between all three regions (*P*=.001). Figures 3a and 3b illustrate the variation of the head circumference in the birth to 3 year age group for boys and girls, respectively, indicating no significant difference between north and central (*P*=.004 for boys and .139 for girls), but a significant difference between north and southwest and central and southwest (*P*=.001). Regional variations in weight for length in the birth to 3 year age group are shown in Figure 4a for boys and Figure 4b for girls with a clearly significant difference between all three regions for boys. For girls, however, the difference was not significant between north and central (*P*=.071), but was significant between north and southwest and central and southwest (*P*=.001).

### Two to 18-years old

**Figure 5:**
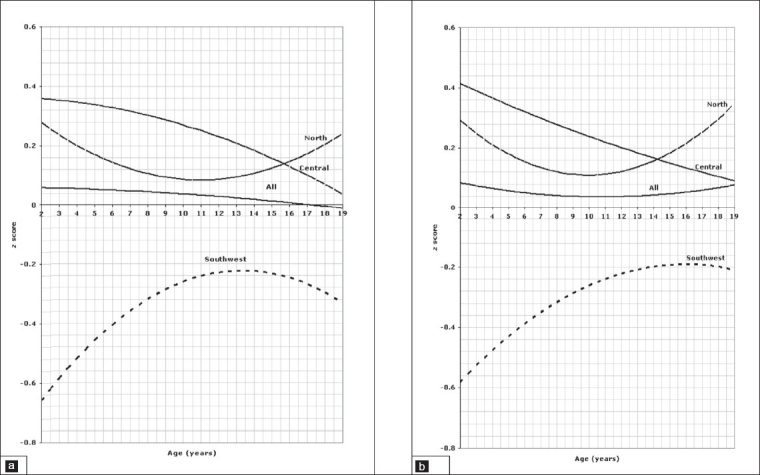
Weight of children aged 2 to 18 years, 5a for boys, 5b for girls.

**Figure 6:**
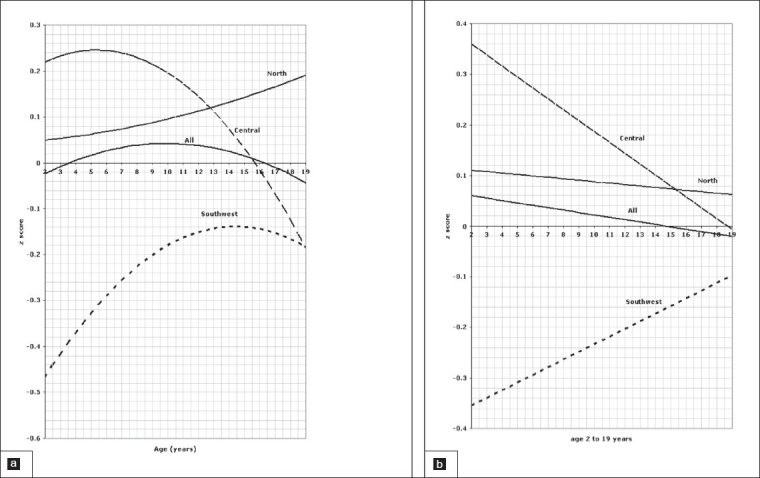
Stature of children aged 2 to 18 years, 6a for boys, 6b for girls.

**Figure 7:**
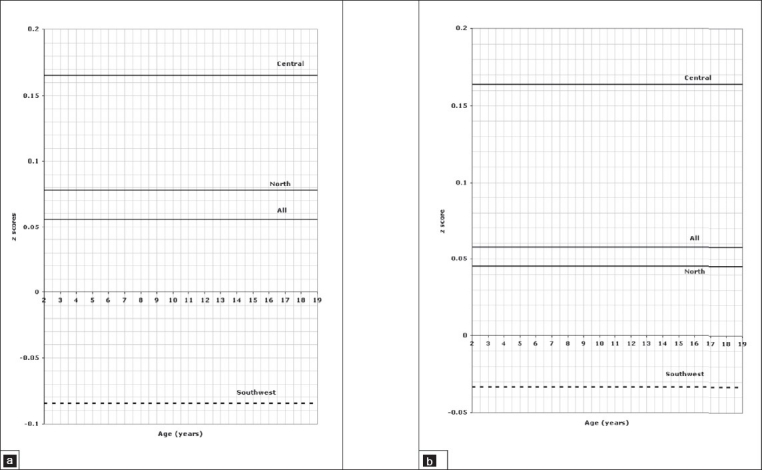
Head circumference of children aged 2 to 18 years, 7a for boys, 7b for girls.

**Figure 8:**
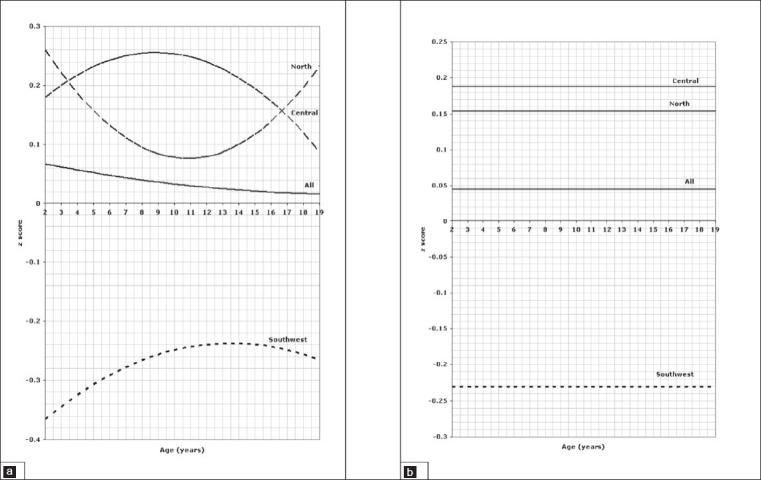
Body mass index of children aged 2 to 18 years, 8a for boys, 8b for girls.

In older children and adolescents from 2 to 18 years of age, comparison of the weight for age z scores is presented in Figure 5a for boys and Figure 5b for girls which shows a significant difference between all three regions (*P*=.001). Regarding stature for age, regional variation is shown in Figure 6a for boys and Figure 6b for girls, again showing statistically significant differences between all regions (*P*=.001). Figures 7a and 7b illustrate the pattern of regional variation of head circumference for age in boys and girls respectively. For boys, there was no significant difference between north and central (*P*=.005), but the difference was significant between north and southwest and central and southwest (*P*=.001). For girls, however the regional variations in head circumference was significant between north and central (*P*=.001) and central and southwest (*P*=.001), but not significant between north and southwest (*P*=.015). The pattern of variation in *body mass index for age* is shown in Figure 8a for boys and Figure 8b for girls. The difference was significant for boys between all three regions (*P*=.001), but not significant for girls between north and southwest (*P*=.272). Tables 1 and 2 show the details of significance of difference between pairs of regions for the age groups birth to 3 and 2 to 18 years, respectively.

**Table 1:** Significance levels of regional variations, 0–3 years.

Parameter	*P* values for boys		
	North-Central	Southwest-Central	Southwest-North
Weight	.001	.001	.001
Length	.001	.001	.001
Weight for length	.003	.001	.001
Head circumference	.004	.001	.001
**Parameter**	***P* values for girls**		
	**North-Central**	**Southwest-Central**	**Southwest-North**
Weight	.001	.001	.001
Length	.001	.001	.001
Weight for length	.071	.001	.001
Head circumference	.139	.001	.001

Number of children: 4331 in central, 1653 in southwestern, and 2099 in the northern region.

**Table 2:** Significance levels of regional variations, 2–18 years.

Parameter	*P* values for boys		
	North-Central	Southwest-Central	Southwest-North
Weight	.001	.001	.001
Stature	.001	.001	.001
Head circumference	.005	.001	.001
Body mass index	.001	.001	.001
**Parameter**	***P* values for girls**		
	**North-Central**	**Southwest-Central**	**Southwest-North**
Weight	.001	.001	.001
Stature	.001	.001	.001
Head circumference	.001	.001	.001
Body mass index	.272	.001	.001

Number of children: 3525 in central, 3413 in southwestern, and 4174 in the northern region.

## DISCUSSION

It is well known that the growth of children as assessed by anthropometric measurements is affected by a combination of genetic and environmental factors. Although studies suggest a minimal role for genetic factors,^13^–^15^ ethnic variations both between individuals and populations cannot be excluded.^16^ Furthermore, variation in growth within the same country is greater than when children from different countries have the opportunity to express their full potential for growth.^17^ However, this ideal situation does not exist in practice because of many factors such as disparity in socioeconomic status, availability of food, access to adequate health services, environmental hygiene, parental educational level and altitude. Regional variation in the degree of development are well known in all countries but are more important in developing countries where more resources are usually assigned to urban than more remote areas. In a study from China, comparing the effect of the reforms on the growth of children in urban and rural areas, it was concluded that despite an overall improvement in child growth during the economic reforms in China, the improvement has not been uniform, as judged by increased differences in height between rural and urban children and increased disparities within rural areas.^18^

In this report, we compare the growth of Saudi children and adolescents between three regions with different population characteristics. The central (Riyadh and Qassim), representing a majority of multiethnic population, three regions from the North (Hail, Jouf, Northern Borders), most likely to have a stable Northern tribal population. Two regions from the southwest (Aseer and Gizan) also most likely to have a majority of stable southern population that is distinct from the north.

The lower growth of children and adolescents in the southwestern population of Saudi Arabia has been assumed, but not documented or quantified. This report demonstrates significantly lower growth of children and adolescents living in southwest regions compared to those in other regions, affecting most growth parameters as indicated by consistently lower z scores. The potential causes of these variations are multiple and many of the well-known factors (environmental and genetic) affecting growth, such as the higher altitude and the predominance of rural settlements in the southwest, may be implicated. The altitude in the city of Abha for example is about 2200 meters above sea level compared to about 700 meters for the north and that of the central region.^19^ In addition, rural settlements account for about 56% and 68% in Aseer and Gizan respectively, compared to about 23% in the north and central regions.^20^ The size of the sample was large and representative, and weighted for the population of each region, making any effect of sample size on the observed differences between regions highly unlikely.

The implication of these differences is considerable and must be taken ino consideration in the assessment of growth of children and adolescents in the southwest who have significantly lower growth parameters than those in the central or northern regions. Further studies focusing on factors associated with lower growth of children in the southwest are needed to make recommendations for improvement in their growth.
